# The impact of aminated surface ligands and silica shells on the stability, uptake, and toxicity of engineered silver nanoparticles

**DOI:** 10.1007/s11051-014-2761-z

**Published:** 2014-12-04

**Authors:** Josephine A. Bonventre, Joseph B. Pryor, Bryan J. Harper, Stacey L. Harper

**Affiliations:** 1 Department of Environmental & Molecular Toxicology, Oregon State University, Corvallis, OR USA; 2 Department of Chemical, Biological, and Environmental Engineering, Oregon State University, Corvallis, OR USA

**Keywords:** Nanomaterials, Silica shell, Surface chemistry, Dispersion, Zebrafish, Environmental and health effects

## Abstract

Inherent nanomaterial characteristics, composition, surface chemistry, and primary particle size, are known to impact particle stability, uptake, and toxicity. Nanocomposites challenge our ability to predict nanoparticle reactivity in biological systems if they are composed of materials with contrasting relative toxicities. We hypothesized that toxicity would be dominated by the nanoparticle surface (shell vs core), and that modulating the surface ligands would have a direct impact on uptake. We exposed developing zebrafish (*Danio rerio*) to a series of ~70 nm amine-terminated silver nanoparticles with silica shells (AgSi NPs) to investigate the relative influence of surface amination, composition, and size on toxicity. Like-sized aminated AgSi and Si NPs were more toxic than paired hydroxyl-terminated nanoparticles; however, both AgSi NPs were more toxic than the Si NPs, indicating a significant contribution of the silver core to the toxicity. Incremental increases in surface amination did not linearly increase uptake and toxicity, but did have a marked impact on dispersion stability. Mass-based exposure metrics initially supported the hypothesis that smaller nanoparticles (20 nm) would be more toxic than larger particles (70 nm). However, surface area-based metrics revealed that toxicity was independent of size. Our studies suggest that nanoparticle surfaces play a critical role in the uptake and toxicity of AgSi NPs, while the impact of size may be a function of the exposure metric used. Overall, uptake and toxicity can be dramatically altered by small changes in surface functionalization or exposure media. Only after understanding the magnitude of these changes, can we begin to understand the biologically available dose following nanoparticle exposure.

**Electronic supplementary material:**

The online version of this article (doi:10.1007/s11051-014-2761-z) contains supplementary material, which is available to authorized users.

## Introduction

The rapid growth of the nanotechnology field will likely result in increased human exposure through both commercial goods and the environment. Various inherent nanoparticle characteristics have been cited as factors driving toxicity, including composition, size, shape, and surface chemistry (Albanese et al. 2012; Nel et al. 2009). Nevertheless, our understanding of how nanoparticles interact with an environment or organism in vivo is still limited in comparison to their abundant use. In addition, as the field advances, more complex composite nanomaterials are emerging in attempts to achieve application-driven needs in healthcare, environmental engineering, and material sciences (Tian et al. 2014; Zhi et al. 2013). Such alterations to bare nanoparticles may have dramatic impacts on their reactivity, stability, uptake, and toxicity (Kasturirangan et al. 2013; Rossi et al. 2010; Sotiriou et al. 2011). Predicting the potential uptake and toxicity of nanocomposites presents a new challenge of whether or not conclusions can be drawn from toxicity studies of single-composition nanoparticle exposures and accurately forecast the potential toxicity of next-generation nanoparticles.

The relative reactivity of the material can be used to predict the toxicity of a nanoparticle, where size, shape, and surface functionalization are comparable. Colloidal silver nanoparticles, for example, are more toxic to developing zebrafish embryos than colloidal gold (Bar-Ilan et al. 2009; Lee et al. 2013). Silver nanoparticles (Ag NPs) are widely studied, largely due to their importance in consumer products, medical devices, and electronics (PEN 2013). The acute toxicity observed with Ag NPs in multiple in vitro and in vivo model systems has been attributed to Ag^+^ released by the particles (Asharani et al. 2011; Choi and Hu 2009; Kashiwada et al. 2012; Park Park et al. 2013a; Powers et al. 2011; Sotiriou and Pratsinis 2010; Yang et al. 2012). Silver ions disrupt membranes, induce reactive oxygen species (ROS) and cytotoxicity, and are generally responsible for the anti-microbial effects of silver (Beer et al. 2012; Gliga et al. 2014; Liu et al. 2010; Park et al. 2011). Simple stabilization by organic molecules, such as phosphate, citrate, and polyvinylpyrrolidone (PVP), has been shown to have differential effects on decreasing the ionic release kinetics (Liu and Hurt 2010; Wang et al. 2014; Yang et al. 2012). The addition of a silica shell is another method utilized to considerably limit the dissolution of Ag^+^ from the core (Bahadur et al. 2011; Choi and Luo 2009; Fuertes et al. 2011; Sotiriou et al. 2011; Ung et al. 1998). Silica-based nanoparticles and silica nanocomposites (i.e., shells), in general, have increased in the material science and biomedical fields largely due to the limited toxicity of silicon dioxide itself, and the ease with which they can be functionalized to alter hydrophobicity or facilitate uptake (Fruijtier-Polloth 2012; Gao et al. 2008; Legrand et al. 2008; Slowing et al. 2006; Suteewong et al. 2011). However, silica-coated titanium dioxide nanoparticles were shown to increase pulmonary inflammation in a rat inhalation model, while neither silica nor titanium dioxide nanoparticles did so alone (Rossi et al. 2010). Given the differential reactivity of silver and silica in biological systems, silica-coated Ag NPs are expected to have reduced toxicity compared to Ag NPs lacking a silica shell (uncoated) due to the reduced ion release from the Ag NP core and the fact that the silica shell, not the silver, would have greater contact with the cell.

Support for this hypothesis stems from multiple studies reporting surface charge as the most common predictive factor driving nanoparticle toxicity (Kim et al. 2013; Li et al. 2013; Liu et al. 2013). The repulsive forces are greater between particles that are charged compared to neutral particles, and therefore charged particles tend to exhibit decreased agglomeration and greater suspension stability, increasing the potential for exposure (El Badawy et al. 2010; Jiang et al. 2009; Suresh et al. 2012). High stability and low agglomeration can promote nanoparticle–cell interactions, enhancing binding and internalization (Nel et al. 2009; Saha et al. 2011; Verma and Stellacci 2010). Positively charged Ag NPs have been shown to be more readily taken up, and exhibit greater toxicity, than comparable anionic or neutrally coated particles in bacteria, daphnids, and zebrafish (Lee et al. 2013; Silva et al. 2014). Silica nanoparticles (Si NPs) and coatings can have a variety of surface functionalization depending the organofunctional alkoxysilanes used, and the magnitude of the surface charge, as measured by zeta potential, can influence the amount and mechanism of uptake harnessed by the nanoparticle to enter cells (Slowing et al. 2006). For example, aminated monodispersed mesoporous Si NPs can be used to transfer DNA plasmids into cancer cells for gene therapy in vitro (Kim et al. 2011). Taken together, this would suggest that the increased presence of a positively charged ligand, like an amine group, on the surface of a silica-coated Ag NP would increase both uptake and toxicity of the nanoparticle.

An additional factor to consider when predicting nanoparticle uptake and toxicity is size. Silver nanoparticles exhibited size-dependent toxicity in the zebrafish embryo, where smaller nanoparticles induced significantly more toxicity than larger nanoparticle at equivalent mass doses (Bar-Ilan et al. 2009). Despite comparable aminated surface functionalization, 75 nm Si NPs were blocked from uptake into human skin cells, while 42 nm Si NPs were not (Rancan et al. 2012). Further, cytotoxicity, resulting from increased ROS production in keratinocytes, was greater for 20 nm Si NPs than for 100 nm Si NPs (Park et al. 2013b). Smaller sizes, and the resulting increase in relative surface area, are generally expected to increase the toxicity of a nanoparticle where surface chemistry and composition are alike.

To investigate the role of core/shell, surface amination, and size on the uptake and toxicity of a composite nanomaterial, we exposed zebrafish embryos to a series of commercially available and custom-synthesized silica-coated silver (AgSi) and Si NPs with amine or hydroxyl termination. The zebrafish embryo model offers a number of advantages for in vivo toxicity screening, including rapid development, non-invasive evaluations of numerous sublethal responses, high fecundity that allows for high-throughput approaches and cost efficiency, and conserved biology with higher vertebrate models (Lele and Krone 1996; Truong and Reif 2014). In *study 1*, aminated or hydroxylated like-sized, commercially available AgSi and Si NPs were compared to address the role of the core and surface amination in driving toxicity. In *studies 2* and *3*, toxicity and uptake were assessed with like-sized custom-synthesized AgSi NPs with three levels of surface amination. Finally, in *study 4*, the impact of size was determined for two comparably aminated AgSi NPs. Our studies address the importance of amine functionalization on dispersion stability in addition to uptake and toxicity, and highlight the use of surface area as a more accurate dose metric to compare across size classes.

## Methods

### Nanoparticles

Ag NPs (20, 70 nm diameter) with silica shells (7, 10, 20 nm thickness) and 80 nm Si NPs with either hydroxyl- or amine-terminated surfaces, phosphate-stabilized (uncoated) BioPure silver spheres (70, 90 nm), and Ag NPs (70 nm) with fluorescein isothiocyanate (FITC) embedded within a 10-nm silica shell (surface enhanced fluorescence, SEF) with varying amination levels were purchased from, or custom synthesized by, nanoComposix (San Diego, CA, USA). Varied-amination levels were based on the recipe used to make commercially available amine-terminated AgSi NPs, and were either half (0.5×), equal (1×), or twice (2×) the amount of (3-aminopropyl)triethoxysilane (APTES) normally used in the synthesis. Detailed nanoparticle specifications and other manufacturer information can be found in Table 1 and Online Resource 1. All nanoparticles were stored at 4 °C until use and handled per manufacturer’s recommendations.

**Table 1:** Nanoparticle descriptions

	Nanoparticle	Abbreviation	TEM-core (nm)^a^	TEM-shell (nm)^a^	Surface chemistry^a^
	BioPure 70 nm silver sphere	70 nm Ag	67.3 ± 5.4	n/a	Phosphate
	BioPure 90 nm silver sphere	90 nm Ag	90.8 ± 7.3	n/a	Phosphate
	Hydroxyl-terminated 70 nm silica-coated silver	Hyd-AgSi	67.7 ± 6.6	19.2	Silica
	Amine-terminated 70 nm silica-coated silver	Amin-AgSi	67.7 ± 6.6	20	Amine-terminated silica
	Hydroxyl-terminated 80 nm silica	Hyd-Si	82.5 ± 5.5	n/a	Silica
	Amine-terminated 80 nm silica	Amin-Si	84 ± 6.9	n/a	Amine-terminated silica
	Amine-terminated silica-coated 70 nm silver, 0.5 × APTES	0.5× (low)	69.9 ± 4.4	9.7	Amine-terminated silica
	Amine-terminated silica-coated 70 nm silver, 1 × APTES	1× or 70 nm (standard)	69.9 ± 4.5	9.2	Amine-terminated silica
	Amine-terminated silica-coated 70 nm silver, 2 × APTES	2× (high)	69.9 ± 4.6	9.9	Amine-terminated silica
	Amine-terminated silica-coated 70 nm silver, 0.5 × APTES, FITC	SEF 0.5×	69.9 ± 4.4	9.7	Amine-terminated silica
	Amine-terminated silica-coated 70 nm silver, 1 × APTES, FITC	SEF 1×	69.9 ± 4.5	9.2	Amine-terminated silica
	Amine-terminated silica-coated 70 nm silver, 2 × APTES, FITC	SEF 2×	69.9 ± 4.6	9.9	Amine-terminated silica
	Amine-terminated silica-coated 20 nm silver, 1×APTES	20 nm 1× (standard)	20 ± 1.8	6.5	Amine-terminated silica

^a^Information provided by manufacturer

### Nanoparticle characterization

The hydrodynamic size and zeta potential for each nanoparticle were measured at 50 μg/ml in Milli-Q (MQ) and fish water (FW, recipe below) using a Malvern Zetasizer Nano (Malvern Instruments Ltd, Worcestershire, UK). Three independent suspensions were run in triplicate to obtain the average hydrodynamic diameter, size distribution, and surface charge measurements in both media types. Nanoparticle tracking analysis (NTA) was performed on MQ suspensions using a Nanosight NS 500 (Nanosight Ltd, Salisbury, UK). Each sample was analyzed in triplicate for mean size and size distribution. X-ray photoelectron spectroscopy (XPS) was used to measure differences in the surface chemistry of the custom-synthesized AgSi NPs using a ThermoScientific ESCALAB 250 X-ray Photoelectron Spectrometer at the CAMCOR Surface Analytical Facility at University of Oregon (Eugene, OR, USA). Atomic composition was averaged from three different areas evaluated on nanoparticle suspensions dried on a chromium-plated grid platform.

### Exposure suspensions

Nanoparticle stock suspensions were obtained monodispersed in MQ water or sodium bicarbonate buffer (Online Resource 2). FW exposure media was prepared by dissolving 0.26 g/l Instant Ocean salts (Aquatic Ecosystems, Apopka, FL) in reverse osmosis water and adjusting the pH to 7.2 ± 0.2 using ~0.01 g sodium bicarbonate (conductivity 480–600 μS/cm). Prior to dilution in FW, stocks were bath sonicated (120 V) using a Fisher Scientific FS30 Ultrasonic Cleaner (without heating) for 25 s to break up agglomerates as per the manufacturer’s recommendation. Serial dilutions were mixed by inverting prior to making the subsequent dilutions. The concentrations used for the exposures were based on preliminary dose responses to a wide range (0.02–250 μg/ml), and based on the total mass of the material provided by nanoComposix. Suspension concentrations used for the commercial and custom-made ~70 nm AgSi and Si NPs were 0.5, 1, 5, 10, 25, 50, and 100 μg/ml; for the bare Ag NPs: 0.02, 0.08, 0.4, 2, 10, 50, and 250 μg/ml; and for the custom-made ~20 nm AgSi NP: 0.1, 0.5, 1, 10, 25, and 50 μg/ml.

### Zebrafish embryo assay

Adult zebrafish (*Danio rerio*) were maintained at the Sinnhuber Aquatic Research Laboratory at Oregon State University. Zebrafish embryos were collected from group spawns of wild-type 5D fish and staged to ensure that all embryos were at the same developmental stage at the start of each experiment (Kimmel et al. 1995). Embryos were dechorionated at 6 h post-fertilization (hpf) with Pronase (Sigma Aldrich) following the procedure of Usenko et al. (2007). At 8 hpf, embryos were exposed to NP suspensions in FW or FW alone (control). Embryos were individually exposed in clear 96-well plates (*N* = 24 per concentration). For commercially available AgSi and Si NPs, a third exposure plate was performed. During waterborne exposures, embryos were incubated at 26.5 °C under a 14:10 h light:dark photoperiod, and observed at 24 and 120 hpf for the appearance of developmental malformations or mortality as described in Truong et al. (2011). Embryos were evaluated at 24 hpf for developmental delay, notochord malformations, spontaneous movement, and embryo viability; and at 120 hpf for malformations of the axis, brain, circulation, eyes, fins, jaw, otic vesicle, pigment, snout, somites, swim bladder or trunk, edema around the heart or yolk sac, and for tactile response.

### Zebrafish embryo uptake assay

Dechorionated embryos were exposed to 50 μg/ml of SEF varied-amine AgSi NPs individually in 96-well plates as described in “Zebrafish Embryo Assay” section. Following exposure, embryos were anesthetized with ethyl 3-aminobenzoate methanesulfonate (Sigma Aldrich) and rinsed with approximately 20 mls FW in groups of 8 embryos to remove loosely associated NPs from the exterior of the animal. Each group of 8 embryos was homogenized in FW (75 μl per embryo, for a total volume of 600 μl), with 3 groups run for each nanoparticle. Control embryos were treated with FW alone. A calibration curve (0–50 μg/ml) was created with each NP in the same concentration of embryo homogenate (75 μl FW/embryo) to control for any background or influence the embryo homogenate, which may have on the nanoparticle UV absorption, and run concurrently with samples. Samples were loaded in triplicate into a 96-well clear-bottom black plate, and scanned on a Molecular Devices (Sunnyvale, CA, USA) SpectraMax M2 spectrofluorometer at the FITC excitation and emission peak wavelengths (490/525 nm, respectively). Fluorescence units were converted to concentrations using the calibration curve. The mass of nanoparticles in embryo homogenate was converted to a concentration per embryo using the average mass of an embryo (1 mg).

### Statistics

Statistical analyses were performed using SigmaPlot 12.2 (Systat Software, San Jose, CA, USA). The presence or absence of developmental endpoints in the embryonic zebrafish assay was analyzed using a Fisher’s Exact test to determine differences between specific treatments and controls. Analysis of variance was used to determine the difference between nanoparticle characteristics in MQ and FW, differences between surface atomic composition measured by XPS, and differences in NP uptake. A significance level of *p* ≤ 0.05 was maintained for all analyses.

## Results

### NP characterization

#### Hydrodynamic diameter

**Fig. 1:**
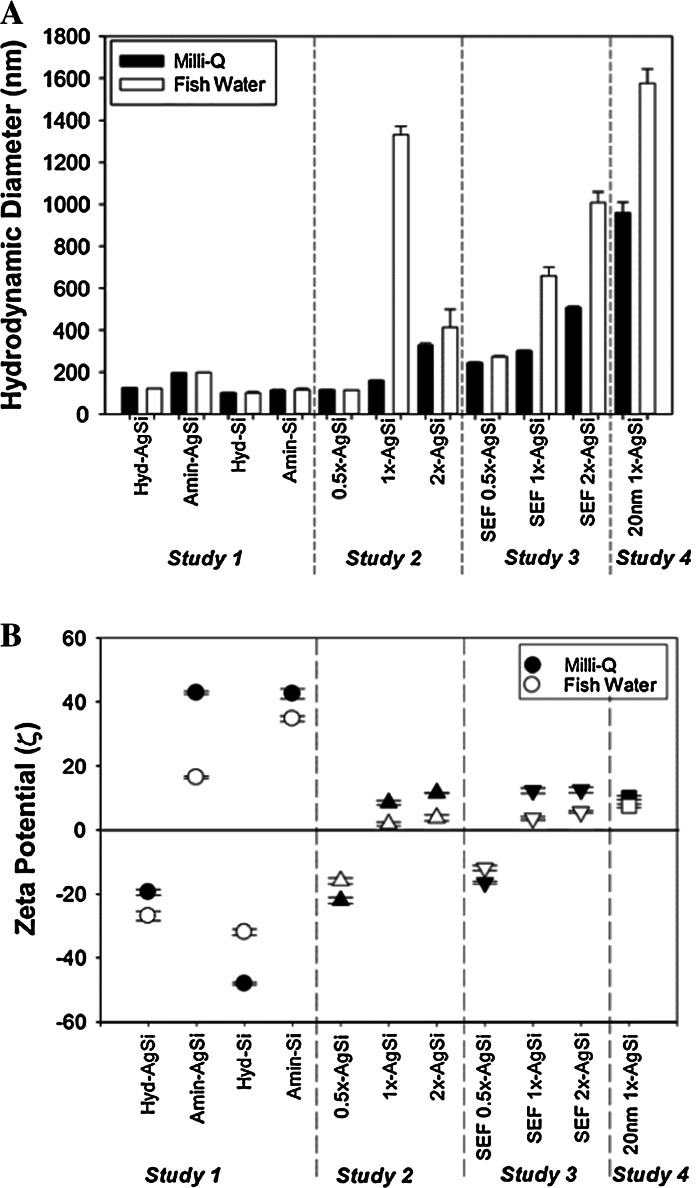
Characterization of NPs with dynamic light scattering and zeta potential. **a** Amination increased the hydrodynamic size of the NPs. The exposure media (fish water, FW) did not alter the hydrodynamic size of the commercially available hydroxyl- or amine-terminated NPs significantly (Study 1) or for 0.5× or 2×, while 1× NPs were significantly larger in the exposure media (Study 2). Hydrodynamic sizes of SEF varied-amination NPs were larger in FW (Study 3). 20 nM 1× AgSi NPs had the largest sizes in both media types (Study 4). **b** Absolute values of zeta potentials of most NPs decreased in FW compared to Mili-Q. 0.5× and SEF-0.5× were negatively charged, while all other amine-terminated NPs were positively charged

Nanoparticles were characterized in both MQ and FW to understand the impact of the exposure media on the suspension stability. The presence of a terminal amine resulted in an increase in hydrodynamic diameter (HDD) compared to paired hydroxyl-terminated or “less aminated” nanoparticles (Fig. 1a; Online Resource 2). For the commercially available NPs (Fig. 1a; Study 1), the amine termination significantly increased HDDs compared to hydroxyl termination. Both AgSi NPs were significantly larger than the Si NPs, even though their primary particle sizes were very similar, ~90 and 80, respectively (Table 1). All four nanoparticles appeared to have low agglomeration in both MQ and FW suspensions, suggesting that the suspensions were stable and well dispersed. Polydispersity index (PDI) was also low, ranging from 0.072 to 0.149 (Online Resource 2B).

For the custom-synthesized varied-amine AgSi NPs, 2× were larger than 1× and 0.5× in MQ, but there was not a significant difference in HDD between 1× and 0.5× (Fig. 1a). The polydispersity indexes for 1× and 2× were larger than 0.5× in MQ, indicating a larger range in sizes was present in suspensions of nanoparticles with increased surface amination (Online Resource 1B). The 1× FW suspensions had significantly larger HDDs than in MQ. The PDI increased from 0.184 in MQ to 0.571 in FW for the 1× NPs. In contrast, 2× suspensions did not exhibit significantly different HDDs in the different media, and only changed from 0.361 to 0.556 (Online Resource 2B, Fig. 1a). The custom-synthesized SEF-AgSi all had significantly larger HDDs in both MQ and FW compared to their non-fluorescent counter parts. The 20 nm 1× AgSi had the largest HDD of all of the NPs measured under both MQ and FW conditions, despite having the smallest primary particle size. The PDI was similar in both MQ (0.483) and FW (0.490) to that of 1× AgSi and 2× AgSi NPs in FW (Online Resource 2B). NTA data largely supported the size data observed with Malvern hydrodynamic sizes, except for the 20 nm 1× AgSi NPs, which recorded a smaller HDD for 20 nm 1× AgSi NPs (Online Resource 2C).

#### Zeta potential

Terminal amines shifted the zeta potential from negative to positive for the commercially available hydroxyl- and amine-terminated AgSi and Si NPs (Fig. 1b). Similarly, the zeta potentials went from negative (0.5×) to positive (1× and 2×) for the custom-made AgSi NPs (Fig. 1b). SEF varied-amine AgSi NPs exhibited similar zeta potentials to their non-SEF counterparts. 20 nm 1× AgSi and 70 nm 1× AgSi exhibited similar zeta potentials in both media, but were significantly lower (absolute value) than commercially available amine-terminated AgSi NPs of Study 1, despite the fact that they were made with similar recipes. Zeta potentials were significantly different in MQ and FW for all of the NPs.

#### X-ray photoelectron spectroscopy analysis

X-ray photoelectron spectroscopy was performed on the custom-synthesized AgSi NPs to confirm the amount of surface nitrogen associated with the different amination levels. As expected, 2× particles had significantly more surface nitrogen (Online Resource 3a); however, there was no significant difference in the percent nitrogen measured on the surface of 1× and 0.5× NPs. Other surface atoms also differed in composition (Online Resource 3b–d). The amount of oxygen on the surface was significantly different for all three NPs, and 0.5× NPs had significantly more carbon than the other two. Finally, there was no significant difference in the amount of silica on the surface, suggesting that the shell was similar between the three varied amine-terminated AgSi NPs.

### Toxicity of AgSi NPs

#### Study 1: surface chemistry and composition

**Fig. 2:**
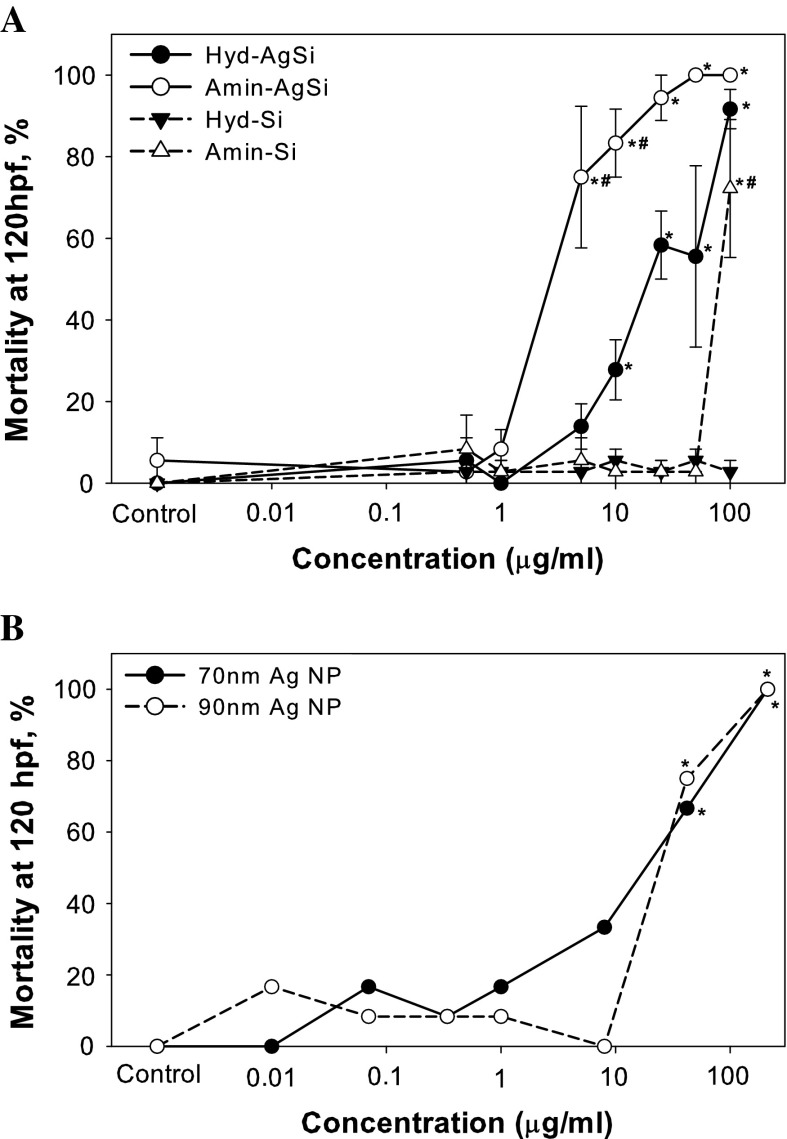
Toxicity of commercially available hydroxyl-, amine-terminated, and phosphate-stabilized NPs. **a** Amine-terminated NPs were significantly more toxic than their hydroxyl-terminated counterparts. Both AgSi NPs were more toxic than the Si NPs. **b** Hydroxyl-terminated AgSi exhibited similar toxicity to comparably sized Biopure AgNP, suggesting that the toxicity may be attributed to the release of Ag ions. *Asterisks* denote significant difference from control, *number sign* indicates significant difference between paired NPs at that concentration

**Fig. 3:**
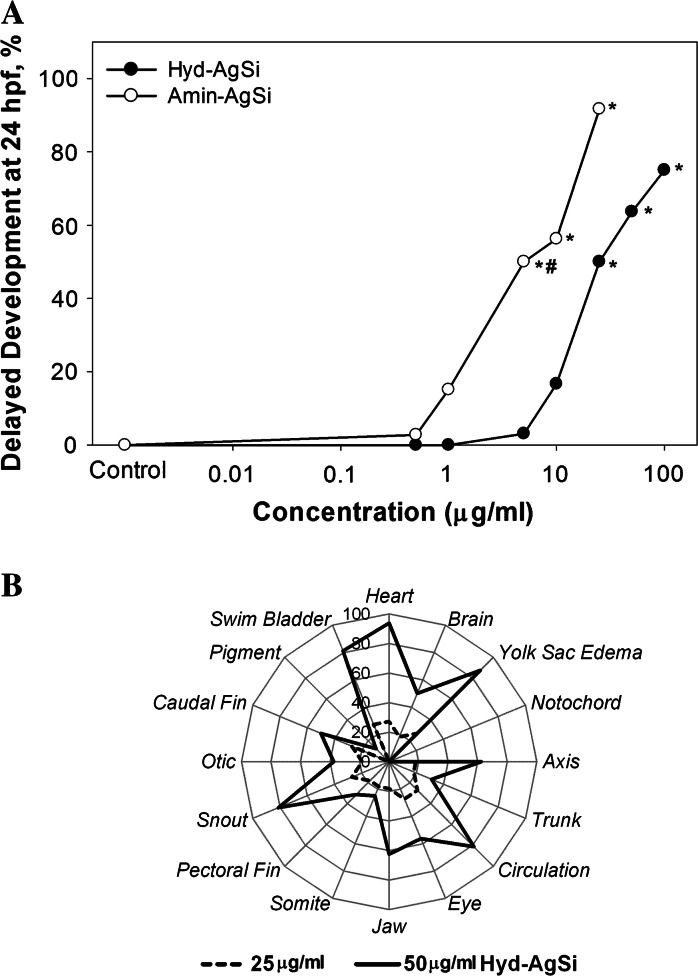
Sublethal effects induced in zebrafish embryos exposed to the hydroxyl- and aminate-terminated AgSi NPs. **a** Both hydroxyl- and amine-terminate AgSi NPs significantly delayed development in a dose-dependent manner at 24 hpf. *Asterisks* denote significant difference from control, *number sign* indicates significant difference between hydroxyl- and amine- AgSi NPs at that concentration. **b** Hydroxyl- AgSi significantly induced a number of malformations at 120 hpf at both 25 and 50 μg/ml. The radar plot shows the differences in toxicity between the two concentrations of hydroxyl- AgSi. All of the sublethal responses represented in the radar plot were significantly different from control, except for pigment and somite malformation

We compared the toxicity of two like-sized AgSi and Si NPs with either hydroxyl- or amine-terminated ligands attached to the surface of silica shells on zebrafish embryo development. In both cases, the amine termination resulted in a significant increase in mortality as compared to the hydroxyl-terminated counterparts (Fig. 2a). The Hyd-AgSi NPs caused significant mortality beginning at 10 μg/ml (Fig. 3a) and resulted in significant increases in malformations and delayed development at 25 μg/ml (Fig. 3b). Embryos exposed to 25 μg/ml exhibited a significant increase in the presence of craniofacial (eyes and snout) and fin (caudal and pectoral) malformations, in addition to pericardial and yolk sac edema. At 50 μg/ml, all of the sublethal responses of the embryonic zebrafish were significantly different from control, except for pigment and somite malformation.

In comparison, Amin-AgSi NPs significantly delayed development at 1 μg/ml, and induced significant mortality at 5 μg/ml, with 100 % mortality observed above 50 μg/ml (Figs. 2, 3a). Delayed development was not observed in the Amin-AgSi at 50 or 100 μg/ml because the treatment induced 100 % mortality by 24 hpf. In contrast to the Hyd-AgSi NPs, lower concentrations of Amin-AgSi did not elicit sublethal toxic effects on the developing zebrafish, other than delayed development. The two surviving fish exposed to 25 μg/ml Amin-AgSi exhibited brain, circulation, eye, fin, jaw, and snout malformation in addition to PE and YSE, but the high mortality resulted in a lack of statistical significance for these end points.

Both Si NPs were significantly less toxic than either of the AgSi NPs. In contrast to the Hyd-AgSi NPs, the Hyd-Si NPs did not induce mortality or morbidity at any concentration tested, while exposure to Amin-Si NPs resulted in a significant increase in mortality at the highest dose tested (100 μg/ml, Fig. 2a). To test the hypothesis that the increase in toxicity between the AgSi and Si NPs was due to the presence of the Ag, zebrafish were exposed to a similar concentration range of non-coated (phosphate stabilized) 70 and 90 nm Ag NPs. Both uncoated Ag NPs induced significant mortality at both 42 ug/ml and 212 μg/ml, compared to control embryos (Fig. 2b). Hyd-AgSi and the uncoated Ag NPs exhibited similar toxicity, while Amin-AgSi NPs were the most toxic particles tested in Study 1.

#### Study 2: varied surface amination and toxicity

**Fig. 4:**
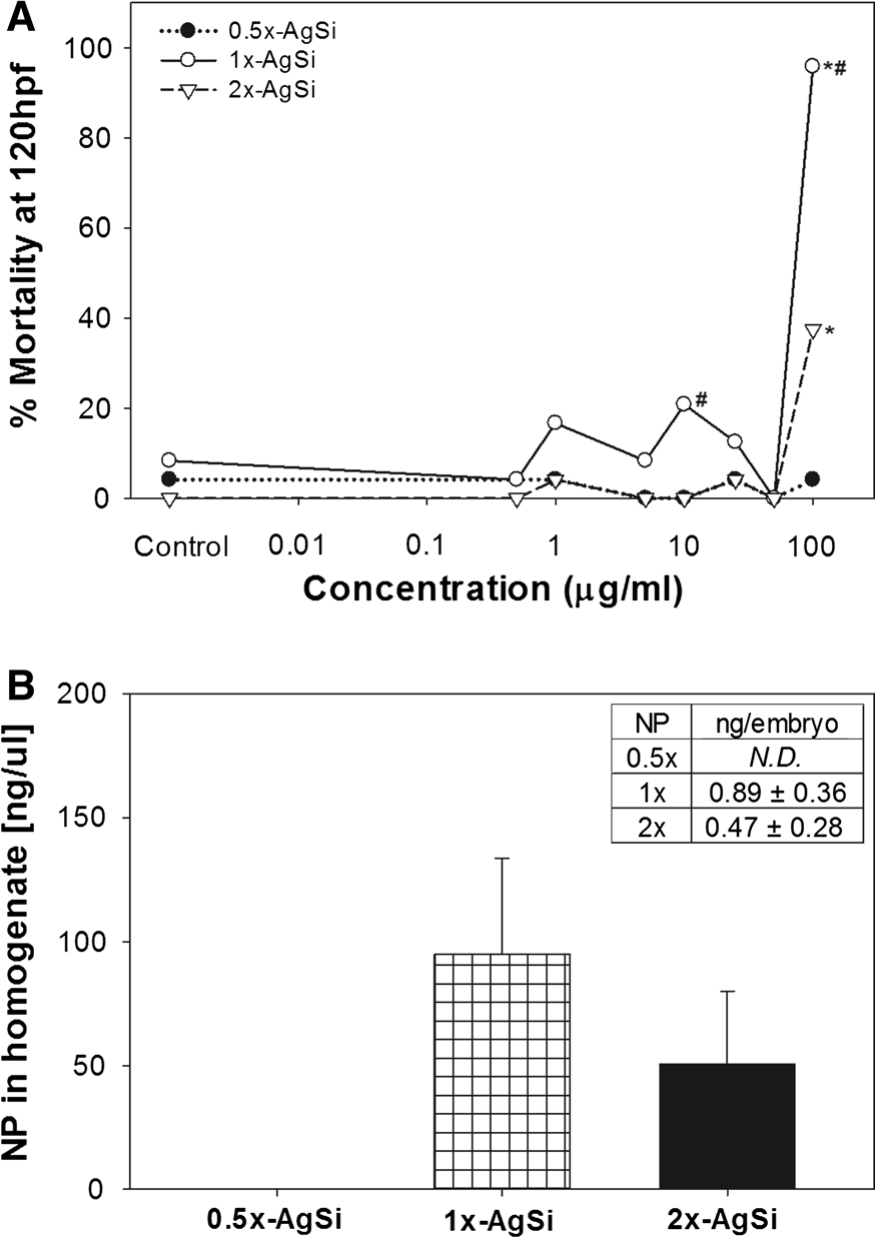
The effect of varying amination AgSi NPs uptake and toxicity. **a** Dose response of varied-amination AgSi NPs. 1× was significantly more toxic than 2× or 0.5×. *Asterisks* denote significant difference from control, *number sign* indicates significant difference between 1× and other NPs at that concentration. **b** Uptake of SEF-AgSi varied-amination NPs. Embryos were exposed to 50 μg/ml of either 0.5×, 1×, or 2× AgSi NPs throughout development. The concentration of 0.5× NPs was below detection. *Inset* table displays the approximate concentration of NP per embryo. n.d. non-detect

Custom-synthesized AgSi NPs with three theoretical levels of amination (0.5×, 1×, and 2×) were used to test the hypothesis that increased amination increases toxicity. AgSi NPs with standard amination (1×) were significantly more toxic than the 0.5× or 2×, inducing 95 % mortality at 100 μg/ml. Low amination level (0.5×) induced no toxicity at the concentrations tested, while high 2× NPs exhibited intermediate toxicity, as it induced mortality in approximately 40 % of the embryos at 100 μg/ml (Fig. 4a). Significant sublethal toxicity was not observed, and the only sublethal endpoint observed with this group of nanoparticles was in the 1 × 100 μg/ml treatment where the only surviving embryo exhibited jaw and caudal fin malformations.

#### Study 3: varied surface amination and uptake

Custom-designed AgSi NPs with surface enhanced fluorescence embedded in the silica shell (SEF) and varying levels of aminated surface ligands (0.5×, 1×, and 2×) were used to test the hypothesis that increased surface amination leads to increased uptake of NPs by embryonic zebrafish. Initial dose response studies demonstrated that the SEF-x particles exhibited similar toxicity to the non-SEF-x particles, with the exception of SEF-2×, which exhibited less toxicity at 100 μg/ml (Online Resource 4a). In a separate, initial range finding study, a concentration of 250 μg/ml induced >95 % mortality in all three SEF-x NPs.

Nanoparticle uptake was measured in embryos exposed to 50 μg/ml of the SEF-0.5×, SEF-1×, or SEF-2× NPs throughout development. The difference in uptake was not significantly different between SEF-1× NPs and SEF-2× NPs, despite almost twice as much SEF-1× NPs was measure in the embryo homogenate compared with SEF-2× (Fig. 4b). The concentration of 0.5× NPs in the embryo homogenate was below the detection limit. On average, each embryo took up approximately 0.89 ng/embryo of the SEF-1×, compared to 0.47 ng/embryo of the SEF-2×.

#### Study 4: the impact of size on the toxicity of AgSi NPs

**Fig. 5:**
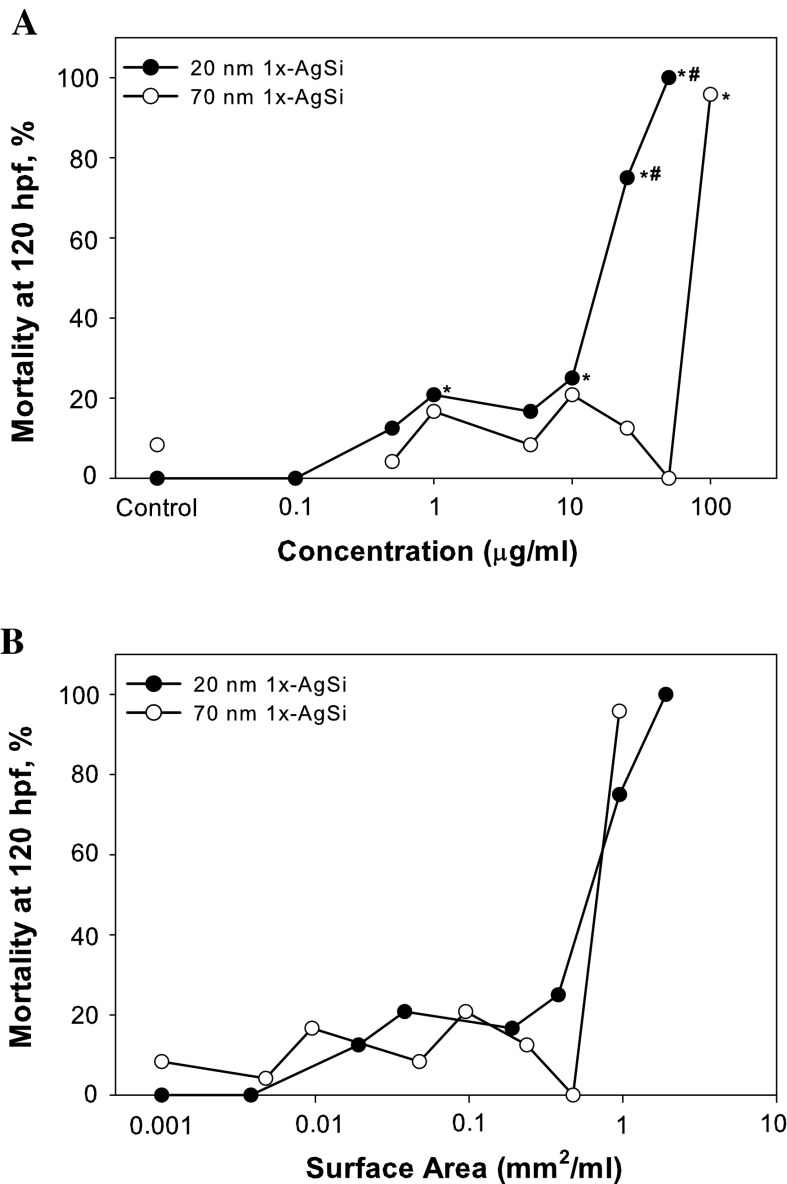
Dose response of 20 or 70 nm standard amination AgSi with mass and surface area-based dose metrics. **a** Mass-based concentration mortality curve demonstrates that 20 nm 1× NPs were more toxic than 70 nm 1× NPs (the data is repeated here for comparison). **b** Estimated total surface area of the NPs per ml of the suspension shows that the toxicity of the two nanoparticles is similar

The effect of size on aminated nanoparticle toxicity was examined by comparing 20 nm to the 70 nm AgSi NPs with comparable (standard, 1×) amination. The 20 nm 1× NPs induced mortality at lower concentrations than was observed with the 70 nm 1×, with a significant increase in toxicity observed at 1 μg/ml in the 20 nm 1×, 100-fold lower than in 70 nm 1× treatments (Fig. 5a). Significant malformations were not observed with 20 nm 1× exposures, similar to the 70 nm 1×. Estimated total nanoparticle surface area was calculated using primary particle sizes, and mass-based concentrations were converted to surface area. The resulting mortality dose responses were similar between 20 and 70 nm AgSi NPs when total estimated surface area was used as the dose metric (Fig. 5b).

## Discussion

Inherent nanoparticle characteristics, like surface functionalization and composition, are important factors in predicting toxicity. As nanotechnology becomes more widespread and the nanomaterials more complex, the need to better understand the impacts of different modifications on nanoparticle reactivity grows. The importance of the nanoparticle surface in driving toxicity has been shown in multiple nanoparticle classes and model systems, including silver (Suresh et al. 2012), latex (des Rieux et al. 2005), nanoceria (Asati et al. 2010), gold (Harper et al. 2011), and dendrimers (Pryor et al. 2014). Here, we tested the hypothesis that the shell and surface ligands were predictive of nanoparticle toxicity in silica-coated (shell) silver nanocomposites. Our studies demonstrated that while both the presence and the amount of surface amination are factors in mediating nanoparticle toxicity, composition of the core also plays a critical role. In contrast, the role of size in nanoparticle toxicity was less important when surface area was applied as a dose metric.

### Dispersion stability is altered by changes in surface functionalization

Dispersion stability affects exposure, while the chemical reactions at a nanoparticle surface ultimately impact the toxicity (Labille and Brant 2010). Therefore, characterizing the stability of nanoparticle suspension is important to interpreting uptake and toxicity data. With the exception of the 20 nm NPs, the primary particle sizes of nanoparticles ranged from 70 to 90 nms (Table 1); however, the HDD and zeta potentials varied greatly with small changes to the overall surface of the nanoparticle. The addition of terminal amines increased the HDD of the nanoparticle and shifted the zeta potential from negative to positive (Fig. 1a, b). Commercially available Amin-AgSi NPs had larger HDDs than Hyd-AgSi NPs, which suggests that the increased HDD observed with custom-made 2× NPs in MQ compared to the 0.5×, and 1× NPs may be the result of more surface amines. The percent nitrogen on the surface of the custom-aminated AgSi NPs ranged from ~3 to 4 %, with 2× having significantly more nitrogen than 1× or 0.5× (Online Resource 3a). Increased surface nitrogen may therefore increase HDD and zeta potential of nanoparticle. Alternatively, the 2× HDD may be larger as a result of an increase in agglomerates, as the stock suspension was not as uniform as 0.5× or 1× stock suspensions. In regards to agglomeration, the 20 nm 1× AgSi Nps were the most prone to agglomeration, as they exhibited the largest HDD of all of the nanoparticles measured under both MQ and FW conditions, despite having the smallest primary particle size. The custom-synthesized NPs may have been more prone to agglomeration than the commercially available NPs, as a result of differences in synthesis, purification, buffers, or other unknown reasons. Differences in agglomeration may account for the differences in toxicity observed between the commercially available and custom-synthesized aminated AgSi NPs (Figs. 2a, 3a). Toxicity of Ag NPs has been shown to correlate to both agglomeration behavior and uptake (Caballero-Díaz et al. 2013).

Overall, the larger sizes observed in the FW suspensions, coupled with the lowered absolute values of zeta potential, indicated that the exposure media altered the stability of the nanoparticle suspension, particularly with the custom-synthesized particles. The destabilization in the exposure media is likely due to the presence of salts in FW interacting with the charged surface molecules (Metin et al. 2011). Zeta potential is a measurement of the electrochemical properties surrounding a nanoparticle, contributing to the repulsive forces that keep the particles suspended and well dispersed (Jiang et al. 2009; Kirby and Hasselbrink 2004; Nel et al. 2009). Since zeta potential is affected by not only the surface charge of the nanoparticle, but also charged species in the media, it was not surprising that zeta potentials were significantly different in MQ and FW for all of the nanoparticles (Fig. 1b). Zeta potentials observed with the commercially available NPs are similar to those previously reported for AgSi NP and Si NP (Bahadur et al. 2011; Duan et al. 2013a, b). The HDD and zeta potential measurements indicated that the suspensions of the commercially available NPs were relatively stable; therefore, the differences in toxicity observed between the four nanoparticles can be attributed to the surface functionalization and composition, not dispersion stability. In contrast, characterizations of the custom-synthesized particles were used to interpret some of the toxicity results. Together, the data demonstrate the importance of using multiple methods of characterizing nanoparticles in order to interpret uptake and toxicity.

### Surface functionalization and composition play a role in nanoparticle toxicity

We hypothesized that the shell and surface ligands would drive the toxicity of a nanoparticle. To support this hypothesis, both commercially available amine-terminated NPs would have had to exhibit similar dose responses in the embryonic zebrafish assay. Instead, while aminated NPs were significantly more toxic than their paired hydroxyl-terminated NPs, both AgSi NPs were more toxic than both Si NPs (Fig. 2a). This, coupled with the similar dose responses between Hyd-AgSi and uncoated AgNPs (Fig. 2b), indicated a significant contribution of the silver to the overall toxicity of the AgSi NPs, despite the presence of the silica shell. Multiple studies have demonstrated that silica shells on silver nanoparticles increase the stability and reduce the release of destructive Ag^+^ (Bahadur et al. 2011; Choi and Luo 2009; Fuertes et al. 2011; Sotiriou et al. 2011; Ung et al. 1998). Ung et al. (1998) demonstrated that thicker the silica shell led to substantially slower dissolution rates, while Fuertes et al. (2011) reported that silica shell largely attenuates the intrinsic toxicity of silver to bacteria cells. Ultimately, the Amin-AgSi NPs were the most toxic nanoparticles tested in Study 1. Together the data indicate that the toxicity observed with Hyd-AgSi is due to the presence of silver, while the toxicity observed with the Amin-AgSi NPs is a combination of both the ionic and amine forces.

Differences in the occurrence of sublethal effects can also be attributed to the surface charge. Hyd-AgSi induced significant increases in malformations and delayed development in addition to mortality at higher concentrations, while Amin-AgSi NPs caused only delayed development and mortality (Fig. 3a, b). Similarly, gold nanoparticles functionalized with cationic trimethylammonium ethanethiol increased mortality in the developing zebrafish more than neutral 2,2-mercaptoethoxyethoxyethanol and 2-,2-mercaptoethoxyethanol, and anionic 2-mercaptoethanesulfonate (MES), while the nanoparticles functionalized with anionic MES induced more sublethal toxicity (Harper et al. 2011). Taken together, the results of Study 1 suggest that while surface amination will enhance the toxicity of a nanoparticle, composition remains a critical factor. It is currently unknown if the influence of amination on toxicity was directly driven by the change in surface charge or by the amines themselves. The pka of APTES is 7.4 (Zhang et al. 1998), which suggests that approximately 50 % of the surface NH_2_-terminated groups will be positively charged in the FW exposure media (pH to 7.2 ± 0.2). While our conclusions can only be directly applied to aminated surface ligands, under our exposure conditions, increasing the amination resulted in increased the positive surface charge (Fig. 1b), and therefore provides support for the hypothesis that increased surface charge leads to increased toxicity.

### Uptake and Toxicity are not linear with increases in surface amination

Our hypothesis that increased amination would result in increased uptake, and toxicity was not supported by Study 2; in that, the toxicity we observed with the custom-synthesized nanoparticles was nonlinear with increased amination (Fig. 4). The toxicity of these NPs also did not follow with the apparent stability of the suspension, based on either visual assessment of sedimentation or the HDD and zeta potential measurements (Fig. 1a, b). All suspensions of the custom-synthesized AgSi NPs appeared well dispersed at the onset of exposure, but settled to the bottom of wells within 24 h. 1**×** suspensions were the most toxic of the three tested in this suite, yet they exhibited stability between that of 0.5× and 2× NPs (visual assessment) and appeared to be the most unstable in the FW exposure media with the largest HDD and smallest absolute value of zeta potential (Fig. 1a, b). 2× suspensions were the first to settle (within 2 h), but were more toxic than 0.5× NPs, which was the most stable of the custom-synthesized AgSi NPs. An alternative explanation for the incongruous toxicity and characterization data is the potential for concentration-dependent agglomeration, observed in previous Ag NPs studies (Wise et al. 2010). HDD and zeta potentials were measured for only the 50 μg/ml suspensions, a concentration at which there was no toxicity observed in any of the varied-amine particles, and may not be representative of what is occurring in 100 ug/ml treatments where differences in toxicity were apparent. In addition, our measurements only provide initial suspension stability and may not accurately describe the suspension over the 5-day experimental period. Future studies should address the balance between an increase in stability afforded by amination, reported in the literature (des Rieux et al. 2005; Kim et al. 2011; Kralj et al. 2012; Slowing et al. 2006; Suteewong et al. 2011; Wu et al. 2013), and an ‘over’ amination, which decreases the stability of the nanoparticles within the exposure media, and therefore the amount of nanoparticles available to interact with the test organisms.

The lack of toxicity observed with the 0.5× NPs, despite the presence of a silver core and negative surface charge, contrasted with our previous conclusions regarding the toxicity observed with the commercially available AgSi NPs. The nanoparticles used in Study 1 were manufactured at a different time, with a different size shell, and stabilized in different buffers and, therefore, cannot be directly compared to those in Study 2. It is possible that the silica shells of the custom-synthesized nanoparticles were better able to reduce the dissolution of Ag ions, as previously reported (Bahadur et al. 2011; Choi and Luo 2009; Sotiriou et al. 2011), resulting in less overall toxicity in Study 2. Despite differences in toxicity and zeta potential between 1× and 0.5×, XPS analysis of did not measure significant differences in nitrogen on the surface (Online Resource 3). The amount of oxygen on the surface was significantly different for all three NPs, and 0.5× NPs had significantly more carbon than the other two, but the role differences in surface oxygen or carbon had on the stability or toxicity of the NPs are unclear.

The uptake data correlated with the toxicity data for the varied-amination AgSi NPs (Fig. 4a, b). The lack of significance between SEF-1× and SEF-2× uptake may be the result of the limited sensitivity of the assay or a small sample size used for the study (*N* = 3). However, together with the toxicity data, these data support our hypothesis that there is a threshold to consider when aminating the surface of a nanoparticle. Our studies were performed in dechorionated embryos to eliminate the chorion as a possible barrier of exposure, as fluorescently labeled Si NPs were previously shown to associate primarily with the chorion (Fent et al. 2010). A complication to using the FITC-labeled SEF NPs was the background level of fluorescence exhibited by zebrafish embryos within the FITC excitation/emission wavelengths, indicating that future studies with SEF particles and zebrafish should consider using alternative fluorescent labels. Finally, the amount of free FITC in solution was presumed to be minimal since the FITC molecules are covalently bonded to the silica shell.

### Impacts of surface amination and size on toxicity

Initially, our data supported the hypothesis that smaller nanoparticles were more toxic than larger nanoparticles with similar surface chemistries (Fig. 5a). The increased toxicity observed with the 20 nm-1× AgSi NPs may be the result of a difference in the efficacy of the silica shell to block Ag^+^ dissolution. Monteiro-Riviere et al. (2013) found that 40 nm AgSi NPs were more prone to breaking down than 120 nm AgSi NPs, as evidenced by empty silica shells and particulates located near nanoparticles in TEM images of cells. This is further evidence that differences in silica shells may not be equal across different groups of nanoparticles, as we observed between Study 1 and Study 2. The thickness of the silica shells varied between the commercially available AgSi NPs and the custom-synthesized NPs (Table 1), but the role played in the nanparticle’s toxicity is unclear, as the commercially available AgSi NPs exhibited the greatest toxicity, but had the thickest silica shells (20 nm thickness). In addition, 20 nm citrate and PVP-stabilized Ag NPs were shown to release more Ag^+^ than similarly coated 110 nm Ag NPs (Wang et al. 2014), which may also contribute to the differences between the 20 and 70 nm-1× AgSi NP toxicity.

Nanoparticle characterization did not help explain differences in toxicity between 20 nm-1× and 70 nm-1× (Fig. 1a, b). HDD and zeta measurements suggested that 20 nm-1× suspensions were less stable (more prone to agglomeration) than the other nanoparticles. However, this observation does not take into consideration the limitations of dynamic light scattering measurements, which are skewed toward the larger particles in suspension. Nanoparticle Tracking Analysis, which uses the Brownian motion of multiple nanoparticle records over a period of time to determine size distribution and mean sizes, showed that the mode size of nanoparticles in the 20 nm-1× suspension was 15 nms, with an average size of 134 nm, indicating that a wide range of sizes were present (Online Resource 1C).

An alternative explanation to the differences observed between the two comparably aminated nanoparticles is that they are a function of the mass-based doses used in the study, which provides only a nominal measurement of the exposure suspension. When mass-based concentrations were converted to surface area of nanoparticles in the suspensions, the dose responses of the 20 nm-1× and 70 nm-1× were more closely aligned (Fig. 5b). Therefore, size was not a factor in driving 20 nm-1× toxicity, rather primary particle surface area of nanoparticle within the suspension was more important, despite the apparent particle agglomeration in exposure solutions. Small particles have the potential for greater surface area at equivalent masses to larger particles, which explains why our hypothesis was initially supported, and may contribute to the number of studies that also report that smaller nanoparticles are more toxic than comparable larger nanoparticles. Yang et al. (2012) demonstrated a relationship between Ag NP toxicity and dissolved silver, but similarly found no correlation between size and toxicity when total reactive surface area was considered. Our study shows that the exposure metric chosen for data analysis can influence the interpretation of the study finding. Even with the assumptions made using primary particle size to estimate total surface area in a suspension, differences between toxicities of the two NPs were small, suggesting that a greater emphasis should be placed on using total reactive surface area within a suspension when comparing the toxicity of different sized NPs with similar composition and surface chemistries.

## Conclusions

The present study characterized the impact of amination, composition, and size on uptake and toxicity of silica-coated (shell) Ag NPs, and possible complications of using any one factor to predict nanocomposite toxicity. Our studies demonstrate that seemingly small changes in surface chemistry of similar nanoparticles can have significant effects on uptake and toxicity. Further elucidation of the effect of amination on biocompatibility and dispersion stability within a given exposure environment is warranted in order to design future nanoparticles with low associated risk. Our studies also highlight the importance of understanding nanoparticle exposure metrics by comparing similarly functionalized nanoparticles at different sizes. Despite the wealth of information demonstrating Ag NP toxicity in multiple models systems, the impact of a silica shell or surface amination on AgNP toxicity is not as well known. There is a need to better understand how small changes in surface chemistry and composition of a nanoparticle can affect overall toxicity in order to better predict biologically relevant exposures.

## Electronic supplementary material

Below is the link to the electronic supplementary material.

Supplementary material 1 (DOCX 17 kb)

Supplementary material 2 (PDF 36 kb)

Supplementary material 3 (PDF 41 kb)

Supplementary material 4 (PDF 116 kb)

Supplementary material 5 (PDF 39 kb)
