# Supplementary material for: The impact of aminated surface ligands and silica shells on the stability, uptake, and toxicity of engineered silver nanoparticles
Source: J Nanopart Res. 2014 Dec 4;16(12):2761. doi: 10.1007/s11051-014-2761-z (PMC4255064; doi:10.1007/s11051-014-2761-z)
Supplement: Supplementary file 1 — Supplementary material 1 (DOCX 17 kb) [file 11051_2014_2761_MOESM1_ESM.docx]

**Supplementary Material Captions:**

**Supplemental Table 1 (ESM_1)** Material Specifications from nanoComposix

**Supplemental Table 2 (ESM_2)** Nanoparticle characterizations in MQ and FW

**Supplemental Fig. 1 (ESM_3)** XPS analysis of custom synthesized AgSi NPs with three levels of amination (0.5x, 1x, 2x).

**Supplemental Fig. 2 (ESM_4)** Dose responses for the SEF particles with varied surface amination.
