# Supplementary material for: The impact of aminated surface ligands and silica shells on the stability, uptake, and toxicity of engineered silver nanoparticles
Source: J Nanopart Res. 2014 Dec 4;16(12):2761. doi: 10.1007/s11051-014-2761-z (PMC4255064; doi:10.1007/s11051-014-2761-z)
Supplement: Supplementary file 2 — Supplementary material 2 (PDF 36 kb) [file 11051_2014_2761_MOESM2_ESM.pdf]

Supplemental Table 1 Material Specifications from nanoComposix

	[Mass]	particles/ml	DLS	Zeta	Solvent	pH	Surface	Lot #
70 nm Silica Coated Silver	1 mg/ml	5.90E+11	133.5	-42.9	MQ	9.2	Hydroxy Terminated Silica	KJW1618A
70 nm Amine-Terminated Silica Coated Silver	1 mg/ml	5.90E+11	217.8	34.8	MQ	8.8	Amine Terminated Silica	KJW1618B
80 nm Non-functionalized Silica	10.8 mg/ml	1.70E+13	126.8	-42.4	MQ	9.2	Hydroxy Terminated Silica	DAC1159
80 nm Amine-Terminated Silica	10.6 mg/ml	1.60E+13	99.2	40.8	MQ	4.7	Amine Terminated Silica	DAC1260
Amine-Terminated Silica Coated Ag70, ½xAPTES	5.0 mg/ml	2.70E+12	115.1	-15	NaBicarb	7.5	Amine Terminated Silica	sso1301-Ag70-½x
Amine-Terminated Silica Coated Ag70, 1xAPTES	5.0 mg/ml	2.70E+12	120.7	-7.4	NaBicarb	7.5	Amine Terminated Silica	sso1301-Ag70-1x
Amine-Terminated Silica Coated Ag70, 2xAPTES	5.0 mg/ml	2.70E+12	364.8	-19.4	NaBicarb	7.5	Amine Terminated Silica	sso1301-Ag70-2x
Amine-Terminated Silica Coated Ag20, 1xAPTES	5.0 mg/ml	1.10E+14	153.4	-24	NaBicarb	7.5	Amine Terminated Silica	sso1301-Ag20-1x
Amine-Terminated Silica Coated Ag70, ½xAPTES, FITC	5.0 mg/ml	2.70E+12	112.8	-16.2	NaBicarb	7.5	Amine Terminated Silica	sso1301-Ag70-½x
Amine-Terminated Silica Coated Ag70, 1xAPTES, FITC	5.0 mg/ml	2.70E+12	121.1	-12.6	NaBicarb	7.5	Amine Terminated Silica	sso1301-Ag70-1x
Amine-Terminated Silica Coated Ag70, 2xAPTES, FITC	5.0 mg/ml	2.70E+12	149.3	-7.85	NaBicarb	7.5	Amine Terminated Silica	sso1301-Ag70-2x
BioPure Silver Nanoparticles, 70nm	0.86 mg/ml	n.r.	72.8	n.r.	phosphate	n.r.	n.a.	CTH1108
BioPure Silver Nanoparticles, 90nm	0.86 mg/ml	n.r.	87.7	n.r.	phosphate	n.r.	n.a.	CTH1112

n.r. not reported, n.a. not applicable
