# Supplementary material for: The impact of aminated surface ligands and silica shells on the stability, uptake, and toxicity of engineered silver nanoparticles
Source: J Nanopart Res. 2014 Dec 4;16(12):2761. doi: 10.1007/s11051-014-2761-z (PMC4255064; doi:10.1007/s11051-014-2761-z)
Supplement: Supplementary file 3 — Supplementary material 3 (PDF 41 kb) [file 11051_2014_2761_MOESM3_ESM.pdf]

Supplemental Table 2 Nanoparticle characterizations in MQ and FW

A Hydrodynamic Diameters of the NPs in different media

Nanoparticle	DLS-MQ	DLS-FW	Zeta-MQ	Zeta-FW
Ag/Si 70/20-0x	125.22 (± 0.8)	122.31 (± 0.25)	-19.45 (± 1.2)	-26.76 (± 2.57)
Ag/Si 70/20-1x	197.1 (± 0.6)	199.0 (± 1.0)	42.8 (± 0.8)	16.6 (± 0.5)
Si 80-0x	101.13 (± 0.11)	101.17 (± 9.93)	-48.07 (± 0.71)	-31.8 (± 1.65)
Si 80-1x	113.19 (± 4.80)	116.41 (± 10.79)	42.53 (± 2.78)	34.93 (± 1.46)
Ag/Si 70/10-½x	115.08 (± 1.5)	114.38 (± 0.14)	-22 (± 1.5)	-15.75 (± 1.34)
Ag/Si 70/10-1x	159.95 (± 2.15)	1083.38 (± 155.83)	8.49 (± 1.17)	2.91 (± 0.80)
Ag/Si 70/10-2x	328.13 (± 15.91)	413.07 (± 149.58)	11.57 (± 0.12)	3.935 (± 1.22)
Ag/Si 70/10-½x_SEF	243.33 (± 5.35)	273.2 (± 4.87)	-16.47 (± 0.67)	-11.7 (± 1.4)
Ag/Si 70/10-1x_SEF	300.2 (± 5.48)	659 (± 73)	12.27 (± 1.45)	3.73 (± 0.78)
Ag/Si 70/10-2x_SEF	507.2 (± 13.17)	1008.87 (± 86.69)	12.53 (± 1.55)	5.74 (± 0.62)
Ag/Si 20/7nm-1x	959 (± 72.69)	1576.8 (± 95.88)	10.03 (± 1.03)	7.61 (± 0.95)

B Average PDIs for the NPs in different media

Nanoparticle	PDI-MQ	PDI-FW
Ag/Si 70/20-0x	0.101	0.083
Ag/Si 70/20-1x	0.149	0.237
Si 80-0x	0.072	0.120
Si 80-1x	0.140	0.163
Ag/Si 70/10-½x	0.089	0.116
Ag/Si 70/10-1x	0.184	0.571
Ag/Si 70/10-2x	0.361	0.556
Ag/Si 70/10-½x_SEF	0.304	0.314
Ag/Si 70/10-1x_SEF	0.325	0.317
Ag/Si 70/10-2x_SEF	0.345	0.436
Ag/Si 20/7nm-1x	0.483	0.490

C NP tracking analysis measurements in MiliQ water

Nanoparticle	Mean	Mode	StDev	10/50/90*
Ag/Si 70/20-0x	115	88	53	58/103/187
Ag/Si 70/20-1x	124	68	76	51/102/227
Si 80-0x	153	85	82	71/138/245
Si 80-1x	144	100	61	76/131/224
Ag/Si 70/10-½x	84	40	55	31/67/152
Ag/Si 70/10-1x	233	325	115	61/257/358
Ag/Si 70/10-2x	281	103	216	101/199/631
Ag/Si 70/10-½x_SEF	135	100	66	71/120/211
Ag/Si 70/10-1x_SEF	300	314	120	149/289/466
Ag/Si 70/10-2x_SEF	429	342	187	231/386/725
Ag/Si 20/7nm-1x	130	15	124	15/74/332

*size at specific percentiles
