# Supplementary material for: The impact of aminated surface ligands and silica shells on the stability, uptake, and toxicity of engineered silver nanoparticles
Source: J Nanopart Res. 2014 Dec 4;16(12):2761. doi: 10.1007/s11051-014-2761-z (PMC4255064; doi:10.1007/s11051-014-2761-z)

Supplemental Fig. 1 XPS analysis of custom synthesized AgSi NPs with three levels of amination (0.5x, 1x, 2x). Differences in the percentages of (a) nitrogen, (b) oxygen, (c) carbon, but not (d) silica were observed. Statistical differences are denoted by letters.

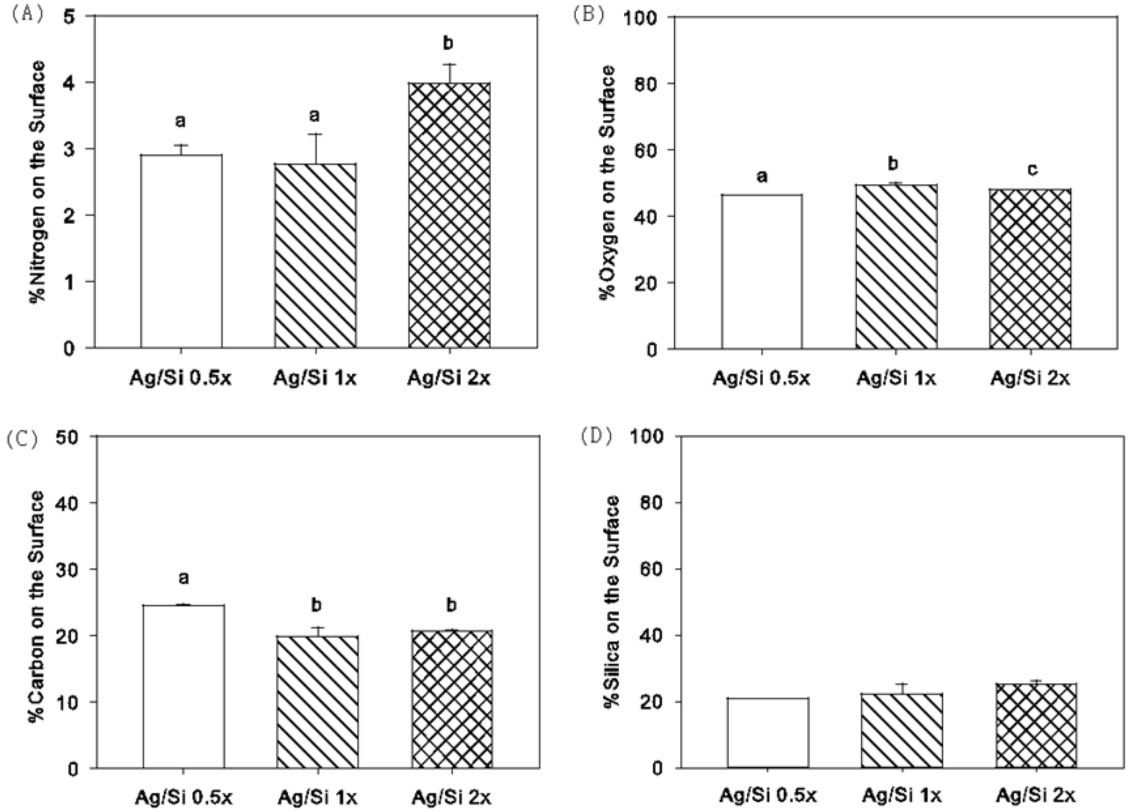

Supplement: Supplementary file 4 — Supplementary material 4 (PDF 116 kb) [file 11051_2014_2761_MOESM4_ESM.pdf]
