# Supplementary material for: The impact of aminated surface ligands and silica shells on the stability, uptake, and toxicity of engineered silver nanoparticles
Source: J Nanopart Res. 2014 Dec 4;16(12):2761. doi: 10.1007/s11051-014-2761-z (PMC4255064; doi:10.1007/s11051-014-2761-z)

Supplemental Fig. 2 Toxicity of SEF particles were similar to their non-SEF counter parts, with the exception of 2x, which showed less toxicity than non-SEF. A separate dose finding study showed 100% mortality for each nanoparticle at 250ppm.

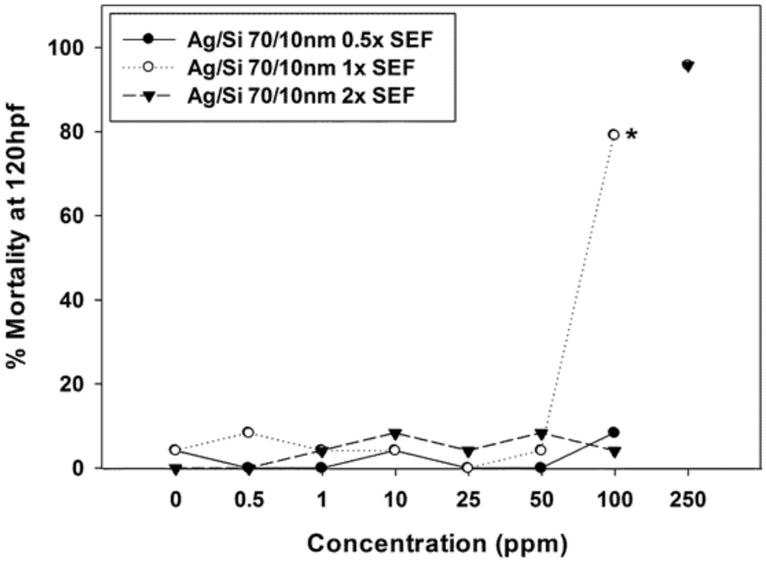

Supplement: Supplementary file 5 — Supplementary material 5 (PDF 39 kb) [file 11051_2014_2761_MOESM5_ESM.pdf]
